# The Birmingham Hospital Saturday Fund

**Published:** 1894-06-02

**Authors:** 


### THE BIRMINGHAM HOSPITAL SATURDAY FUND.

Sir,—We are the surviving members of the committees which worked with the late Mr. Sampson Gamgee in the Working Men's Fund for the extension of the Queen's Hospital and the establishment of the Hospital Saturday movement. We have read your article on the Birmingham Fund in The Hospital of the 26th inst., and also the reply which Mr. Smedley is sending to you. From our personal knowledge we know that Mr. Smedley's statements as to the history of the movement, and Mr. Gamgee's attitude to the weekly system of contributing, which is the foundation of the success at the present time, are absolutely correct. Mr. Gamgee was wedded to the idea of Hospital Saturday being a day of labour sacrifice, and this was the foundation of the movement which he established. He strongly advised against our abandoning this as the method of collecting and substituting for it the weekly method of contributing. It is within our knowledge that neither Mr. G. F. Muntz nor Mr. George Dawson had anything to do with the establishment of the movement, except that after the first collection the former gave £500 towards the expenses of the same year. We do not desire to depreciate Mr. Gamgee's great services in the founding of the Fund, and we cherish for his memory the truest affection, but we feel bound to make this statement in defence of the truth.—We remain, yours obediently,

Henry Allwood.
George Cashmore.
Richard Rogers.
Henry Hughes.
William Gilliver.

*** Mr. Gamgee's view of the weekly system we gave last week in Mr. Gamgee's own words. We have received a telegram from Mr. Smedley in reference to the reply alluded to in the above letter, in which he says, "Just received telegram; impossible to revise to-night." So we shall hope to publish it next week.—Ed. The Hospital.

